# Tuning Surface Molecular Design of Porous Carbon for Blue Energy Harvesting

**DOI:** 10.34133/research.0173

**Published:** 2023-06-19

**Authors:** Jian Yu, Zhong-Lin Wang, Tianwei Ma

**Affiliations:** ^1^ Department of Civil and Environmental Engineering, University of Hawaii at Mānoa, Honolulu, HI 96822, USA.; ^2^ Beijing Institute of Nanoenergy and Nanosystems, Chinese Academy of Sciences, Beijing 101400, China.; ^3^ College of Engineering, Texas A&M University-Corpus Christi, Corpus Christi, TX 78412, USA.

## Abstract

Capacitive mixing is a promising blue energy technology due to its membrane-free electricity generation and long electrode life cycle. However, because of limited performance, existing systems do not lend themselves to practical implementation. Although it is a crucial factor directly influencing electrode behavior, surface chemistry has largely been overlooked in capacitive mixing. Here, we show that manipulating surface functionalization alone can tune the responses of electrodes to produce a high voltage rise without altering the pore structure of the electrodes. Our findings reveal that the spontaneous electrode potential of a surface-modified carbon electrode shifts negatively proportional to the surface charge due to the surface groups, which explains why and how manipulating the surface chemistry can improve the power generation capacity. Using electrodes fabricated with identical activated carbon material but with different surface treatments, we have achieved a remarkably high power density of 166 mW/m^2^ delivered to an electrical load under a 0.6 M to 0.01 M salinity gradient, with the total power generated of 225 mW/m^2^. The corresponding volumetric power densities were 0.88 kW/m^3^ net and 1.17 kW/m^3^ total. The volumetric power density of our prototype is comparable to or better than those of prevailing membrane technologies, such as pressure retarded osmosis and reverse electrolysis, whose volumetric power density values are 1.1 kW/m^3^ and 0.16 kW/m^3^, respectively. In the seawater stage, the net power density reached 432 mW/m^2^ or 2.3 kW/m^3^. Such performance far exceeds existing membrane-free systems, with the highest reported power density of 65 mW/m^2^ under a 0.5 M to 0.02 M salinity gradient (121 mW/m^2^ in this work). The device demonstrated unparalleled durability, maintaining 90% of the maximum energy capacity after 54,000 charge–discharge cycles.

## Introduction

Blue energy, which refers to the good amount of Gibbs free energy released when solutions of different salt concentrations mix, has attracted increasing attention [1–6]. It is estimated that 1,700 TWh of energy is abundantly available from the natural mixing of river water and seawater each year. Unlike solar and wind, blue energy can be harvested under almost all weather conditions, which renders it a reliable, renewable energy source.

Pressure retarded osmosis (PRO) [1,7,8] and reverse electrodialysis (RED) [9–12] are by far the two most studied methods for blue energy harvesting. The PRO approach utilizes semipermeable membranes to create an osmotic pressure difference to drive a turbine for electricity generation while the RED approach uses ion-selective membranes to create an ionic flux, which facilitates electricity generation via redox reactions at the electrodes. Despite decades of development, these methods still do not meet the requirements for practical deployment mainly due to the limited performance of the membranes [13]. There is a renewed interest in RED lately as membranes containing nanopores may improve the power density considerably. However, the demonstrated improvements were based on a single-nanopore scenario [4,14–23] or a micro-sized (e.g., 0.0625 mm^2^) membrane [5]. Whether a macro-scale membrane incorporated with a large number of nanopores will perform desirably is questionable [24].

Capacitive mixing harvests blue energy based on the change of the electric double layer (EDL) at the electrode–electrolyte interface in response to a salinity gradient [25–32]. It does not require membranes or chemical reactions for power generation. Although much effort has been devoted to this technology, the performance of existing systems is still not economically competitive—the gain from eliminating membranes remains hypothetical [2,8]. Battery mixing—a concept that appears to be closer to capacitive mixing than any other technology—has been proposed to improve power density. Still, they require battery-type electrode reactions or membranes to operate [33–35]. Therefore, the performance improvement is at the cost of the cycle life of electrodes or reintroducing membranes. The prospect of success of battery mixing is debatable at best.

Capacitive systems have traditionally been designed by focusing solely on the electrolyte side of the interface, specifically the EDL [25,26,32]. The capacitance change induced by the salinity gradient is the only factor considered in capacitive mixing systems. It has been shown that surface functional groups can be utilized to improve capacitive mixing as they can shift the spontaneous electrode potential [29,36]. However, the mechanism by which surface groups shift the electrode potential is poorly understood. Here, we present a theoretical model that considers both sides of the electrode–electrolyte interface, thereby explaining how and why surface groups shift the spontaneous electrode potential. Our model shows that the electrode potential results from two competing mechanisms, i.e., the interfacial potential jump originating from the surface dipoles [37–39] and the Volta potential [40,41] of the EDL. The extractable portion of the Gibbs free energy is proportional not only to the capacitance change induced by mixing but also to the square of the combined interfacial potential jump of the electrodes. We have found that the electrode interfacial potential jump is responsible for the direction of the shift in the spontaneous electrode potential. Because of the interfacial potential jump, oxygen-containing functional groups induce a positive electrode potential shift while creating a negatively charged surface due to deprotonation. Conversely, nitrogen-containing functional groups shift the electrode potential more negatively while the induced surface charge is positive due to protonation. If two such carbon electrodes in the same bulk solution are brought to the same surface potential via circuit connection, they will acquire different amounts of surface charge. Therefore, by manipulating the surface functionalization levels of the electrodes alone, one can tune the system response to optimize the energy harvesting performance. Using electrodes fabricated with identical activated carbon material but with different surface treatments, we have achieved a remarkably high power density of 166 mW/m^2^ delivered to an electrical load under a 0.6 M to 0.01 M salinity gradient and 121 mW/m^2^ under a 0.5 M to 0.02 M salinity gradient, far exceeding the highest value reported in the literature [29,42]. Under a 0.6 M to 0.01 M salinity gradient, the power density for the seawater stage reached 432 mW/m^2^, with the total power extracted (including that consumed by the internal resistance) being 480 mW/m^2^. The volumetric power density of our system is 0.88 kW/m^3^. Our device has demonstrated unparalleled durability, maintaining 90% of the maximum energy capacity after 54,000 charge–discharge cycles. Due to the versatility of surface treatment processes, we anticipate this work will help open doors to new avenues for capacitive mixing that will lead to blue energy harvesting systems suitable for large-scale implementations.

## Results

### Electrode potential

Contact electrification is a universal phenomenon that occurs at the interface of two phases due to a number of mechanisms [43–46]. When an electrically neutral electrode, fabricated with high-purity carbon with surface functionalization, is immersed in an aqueous solution, protonation or deprotonation of the surface functional groups [43] is the dominant mechanism leading to the establishment of an EDL at the electrode–electrolyte interface. The capacitance of the EDL, *C*, may be modeled as that of two capacitors connected in series, i.e., $\frac{1}{C}=\frac{1}{C_{\mathrm{st}}}+\frac{1}{C_{d}}$, where the capacitance of the Stern layer, *C*_st_, depends on the sizes of the ions in the solution, independent of the concentration of the solution. The capacitance of the diffuse layer, *C*_d_, increases as the concentration of the electrolyte increases [47,48].

The electrical potential on the electrolyte side of the interface (relative to the potential of the bulk solution) is the so-called Volta potential, *ψ*_0_ [39–41]. According to the Gouy–Chapman–Stern theory, *ψ*_0_ = *σ*_0_/*C*_st_ + *ψ*_d_, where *σ*_0_ is the total surface charge of the electrode, and *ψ*_d_ is the potential at the plane where the diffuse layer of the EDL starts and extends into the bulk. As a first principal approximation, *ψ*_d_ = *σ*_0_/*C*_d_. Because a higher concentration results in a larger total capacitance of the EDL, *ψ*_0_ decreases as the concentration increases. The Volta potential, *ψ*_0_, is not measurable [37,49,50], but using a reference electrode, one can measure the electrode potential, *U*_0_ (Fig. S1), as

$$U_{0}=\chi +\psi_{0}+U_{r}$$ (1)

where *χ* is the interfacial potential jump determined by the total dipole moment at the interface [38,51,52] and *U*_r_ accounts for the sum of other interface potential differences, e.g., the effect of the reference electrode. When the electric charge of surface dipoles on the carbon electrode side is negative, *χ* is negative, representing a potential drop from the electrolyte to the electrode side, which results in a lower *U*_0_ (Fig. S2A). Conversely, *χ* is positive when the charge of surface dipoles on the carbon electrode side is positive, leading to a higher *U*_0_ (Fig. S2B).

**Fig. 1.**
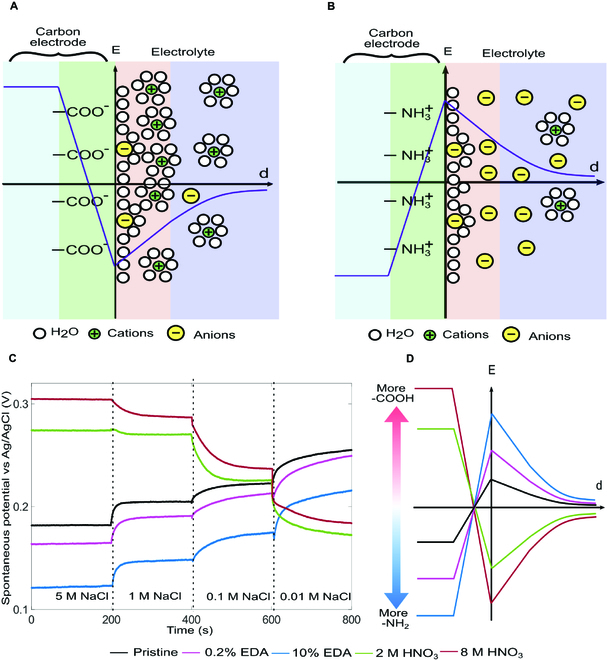
Electrode potential establishment of a carbon electrode in an aqueous solution. (A) A schematic of a carbon–electrolyte interface with predominantly negatively charged surface functional groups. (B) A schematic of a carbon–electrolyte interface with predominantly positively charged surface functional groups. (C) Time histories of potentials of surface-modified YP80F electrodes (versus Ag/AgCl) under various salinity gradients. (D) A schematic of electrical potential profile from the bulk electrode to the bulk solution for electrodes with different levels of functionalization under the same salinity condition.

Figure 1 schematically shows the distribution of electric potential from the bulk of the electrode to the bulk solution and the measured electrode potentials under various electrolyte concentrations. Figure 1A illustrates the case of an electrode with oxygen-containing functional groups (e.g., -COOH, -OH). The surface dipoles (C^+^−O^−^) [53] (Fig. S2A) generate a potential jump (*χ*) from the electrolyte to the electrode at the interface. Meanwhile, the surface groups tend to deprotonate (e.g., -COOH→−COO^−^+H^+^), resulting in a negative *ψ*_0_. The measured electrode potential *U*_0_ is the result of these two competing mechanisms. Figure 1B shows the scenario where nitrogen-containing functional groups (e.g., -NH_2_) are attached to the carbon surface (Fig. S2B). The surface dipoles (N^−^− H^+^) result in a potential drop from the electrolyte to the electrode, while *ψ*_0_ is positive because of protonation of the functional groups (e.g., $-NH_{2}+H^{+}\to -NH_{3}^{+}$) [54]. Similar to the case of oxygen-containing surface groups, the interfacial surface jump (*χ*) and the Volta potential (*ψ*_0_) contribute to the electrode potential in an opposite way. Because it results solely from surface dipoles, *χ* is independent of the concentration of the solution. Therefore, the electrode potential of an electrode with oxygen-containing surface groups reduces with a decreasing concentration because the Volta potential becomes more negative in a lower concentration. The trend is the opposite for electrodes with nitrogen-containing surface groups due to the positive Volta potentials. We have used commercial materials, i.e., activated carbons YP80F and YP50F, to fabricate electrodes with different levels of functionalization and measured the electrode potentials in NaCl solutions of various concentrations. We considered two functional groups, i.e., the carboxyl and amine groups. The carboxyl groups were grafted to the surfaces of carbon particles by oxidizing the carbon materials with nitric acid. In contrast, the amine groups were attached to the carbon surface through physical adsorption of the ethylenediamine (EDA) reagent. The electrodes were immersed sequentially in 5 M → 1 M → 0.1 M → 0.01 M solutions for 200 s each. The results shown in Fig. 1C suggest that the spontaneous electrode potential decreases (increases) as the concentration decreases if an electrode is functionalized with oxygen-containing (nitrogen-containing) groups. Similar results were obtained using activated carbon YP50F (Fig. S6). A higher amount of polar functional groups leads to a higher value of surface charge and, in turn, results in a higher voltage rise for a given salinity gradient. The surface charge for each electrode varies due to the different amounts of functional groups, with *σ*_0_ (10 % EDA) > *σ*_0_ (0.2 % EDA) > *σ*_0_ (pristine) > 0 > *σ*_0_ (2 M HNO_3_) > *σ*_0_ (8 M HNO_3_). The corresponding voltage rise follows the same pattern. The observation is consistent with the results from potentiometric acid–base titration, suggesting that the oxygen-containing (nitrogen-containing) surface groups result in a negative (positive) surface. The potentials of electrodes with more oxygen-containing (nitrogen-containing) surface groups were higher (lower) than electrodes with fewer surface groups in all concentration levels, indicating a strong correlation between the number of surface groups and the interfacial jump, *χ*, as schematically shown in Fig. 1D.

### Surface properties of treated carbons

**Fig. 2.**
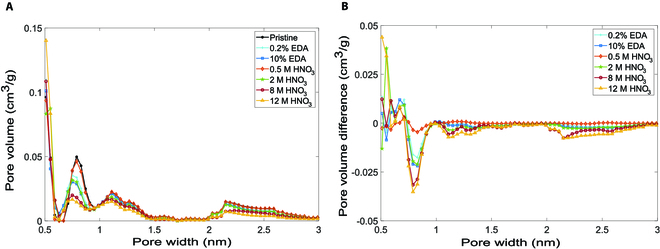
Pore characteristics of surface-modified YP80F. (A) Pore size distributions. (B) Pore volume differences between surface-modified and pristine YP80F.

We introduced two types of surface treatments to add surface groups. Oxyen-containing groups were added through oxidation with nitric acid, while nitrogen-containing functional groups, i.e., amine groups, were adsorbed onto the carbon surface using the EDA reagent. Our results (Fig. 2 and Figs. S3 and S4) indicate that both treatments did not noticeably alter the particle size but moderately changed the pore size distribution. The addition of surface groups led to a shift in the distribution of the pore widths below 1 nm to a lower pore-width range. For instance, as shown in Fig. 2, a portion of YP80F pores of 0.7 to 0.9 nm was shifted to 0.6 to 0.7 nm, and a larger portion of these pores was shifted as the number of surface groups increased. Pore shrinkage caused by the shift in pore size distribution reduced the surface areas. It also slightly decreased the micropore and mesopore volumes (and hence the total pore volume), reducing porosity, as shown in Table and Table S4. Interestingly, the reduction in surface area was greater than that in porosity. For example, we observed a 16% reduction in surface area with 10% EDA, while the corresponding reduction in porosity was 7%. The same trend was observed for YP50F.

**Table.** Material properties of activated carbon YP80F with different surface modifications.

Carbon	BET area (m^2^/g)	Porosity (%)	Capacitance (F/g)		Element wt%		
			0.6 M	0.01 M	C	N	O
Pristine YP80F	2,010.8	67.6	38.0	15.6	98.4	0	1.6
YP80F 0.2% EDA	1,835.1	64.7	36.4	13.9	97.12	0.45	2.43
YP80F 10% EDA	1,695.8	62.7	34.4	11.5	95.88	1.66	2.46
YP80F 0.5 M HNO _3_	1,957.1	66.7	45.5	19.9	94.95	0	5.05
YP80F 2 M HNO _3_	1,779.0	64.9	52.5	18.1	91.46	0	8.54
YP80F 8 M HNO _3_	1,588.5	64.9	64.8	17.8	86.9	0	13.1
YP80F 12 M HNO _3_	1,498.5	61.2	42.2	14.7	81.9	0	18.1

Activated carbon materials, such as those used in this study, offer several advantages over other carbon materials for harvesting energy from salinity gradients. With over 2,000 m^2^/g surface area, activated carbon YP80F has more than double the surface area of other carbon materials, including carbon nanotube powders (100 to 1,000 m^2^/g), 3D carbon nanotube forest (1,000 m^2^/g) [55], and carbon black (75 m^2^/g), making it possible to achieve exceptionally large energy capacity. Additionally, the activated carbon material used in this study has pores that are more concentrated in the micropore range (0.5 to 3 nm) compared to other carbon materials reported in the literature [32,55]. This concentration of micropores enhances the voltage rise response, which is critical for generating a high power density from salinity gradients. Furthermore, the activated carbon materials used in this research are easier to incorporate with the binders to fabricate thicker electrodes than single-walled carbon nanotubes and, thus, further increase the energy density of the device.

### Surface charge and voltage rise

**Fig. 3.**
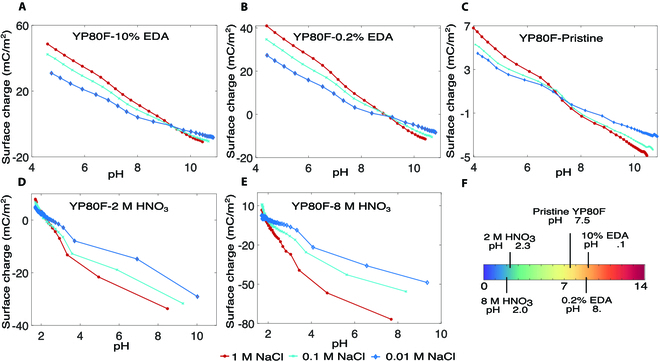
Surface charge of treated and pristine YP80F obtained by potentiometric acid–base titration in sodium chloride solutions. Surface charge of YP80F corresponding to (A) 10% EDA treatment, (B) 0.2% EDA treatment, (C) no surface treatment, (D) 2 M HNO_3_, and (E) 8 M HNO_3_. (F) Points of zero proton charge (PZPC).

Two main sources contribute to the total surface charge (*σ*_0_) of a high-purity carbon immersed in an aqueous solution, i.e., *σ*_0_ = *σ*_e_ + *σ*_H_, where *σ*_e_ represents the surface charge (e.g., free electrons) from an external source, and *σ*_H_ represents the proton charge of the surface groups. If the electrode of concern is polarizable, *σ*_e_ is independent of the concentration, whereas *σ*_H_ is concentration-dependent. We have performed potentiometric acid–base titration [56,57] to determine the proton charge of the pristine and the surface-modified YP80F in 3 NaCl solutions, i.e., 1 M, 0.1 M, and 0.01 M. As shown in Fig. 3C, the points of zero proton charge (PZPC) of all YP80F materials did not change with the concentration, indicating the absence of specific ion adsorption. A higher concentration resulted in a higher level of surface proton charge under a pH away from the PZPC. The pristine material was neither functionalized nor charged by an external source. A PZPC of approximately 7 (Fig. 3C) indicates that the surface charge was proton charge only. As expected, the presence of carboxyl groups lowered the PZPC due to the deprotonation of carboxyl in NaCl solutions, whereas amine groups increased the PZPC due to their tendency to protonate. A higher level of functionalization resulted in a higher level of *σ*_H_ as evidenced by the more substantial deviation of the PZPC from that of the pristine material (Fig. 3F). Overall, the results from the titration experiments validate our model shown in Fig. 1. Therefore, a functionalized polarizable electrode responds to a concentration gradient through two mechanisms. A decrease in concentration reduces the EDL capacitance and, thus, increases the Volta potential (*ψ*_0_), but at the same time lowers the surface charge by decreasing *σ*_H_, thereby decreasing the Volta potential. The net potential rise of the electrode (Δ*U*_0_) when the concentration is changed from *c*^a^ and *c*^o^ can be expressed as

$$\Delta U_{0}=k\left(U_{0}^{a}-\Phi \right)$$ (2)

where $k=\left[\alpha \eta_{1}\left(\eta_{1}+\eta_{2}\right)/\left(1+\eta_{2}\right)-1\right],\eta_{1}=C_{d}^{a}/C_{d}^{o},\eta_{2}=C_{d}^{a}/C_{\mathrm{st}}$ . Superscripts “a” and “o” represent the quantities corresponding to the indicated concentrations, respectively, Φ = *χ* + *U*_r_, and *α* represents the effect of concentration on the total surface charge, i.e., $\alpha =\sigma_{0}^{o}/\sigma_{0}^{a}$, where $\sigma_{0}^{a}=\sigma_{e}+\sigma_{H}^{a}$ and $\sigma_{0}^{o}=\sigma_{e}+\beta \sigma_{H}^{a}$, with *β* being a non-dimensional coefficient determined by the concentrations (*c*^a^ and *c*^o^). Note that $U_{0}^{a}=\Phi$ leads to a zero potential rise, indicating the absence of an EDL.

**Fig. 4.**
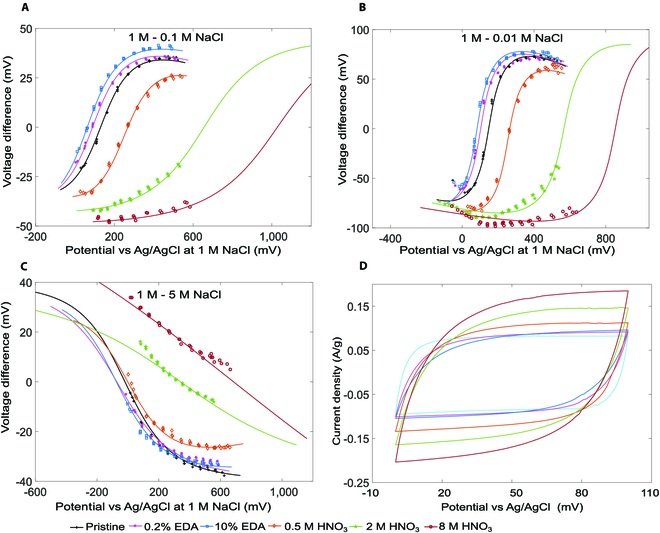
Results obtained from voltage rise and cyclic voltammetry experiments for YP80F electrodes. (A to C) Voltage rises for 1 M→0.1 M, 1 M→0.01 M, and 1 M→5 M, respectively. (D) Cyclic voltammetry profiles (2 mV/s) of YP80F electrodes in 0.6 M NaCl. In (A) to (C), markers: experimental data, lines: Gouy–Champman–Stern model.

Figure 4 shows the responses of electrodes fabricated with pristine and surface-modified YP80F, subjected to salinity gradients. We have used a 3-electrode setup for the experiments. In the experiments, we first applied an electric potential ($U_{0}^{a}$) to the working electrode (YP80F) in a 1 M NaCl solution against the counter electrode (Pt) and measured the electrode potential using an Ag/AgCl reference electrode. We then changed the solution and measured the electrode potential ($U_{0}^{o}$) again to calculate the voltage rise ($\Delta U_{0}=U_{0}^{o}-U_{0}^{a}$). As shown, the experimental data fit the Gouy–Chapman–Stern model well—a much-expected result because the voltage rise is determined by the EDL. According to Eq. 2, $\Phi =U_{0}^{a}$ when Δ*U*_0_ = 0. Nitrogen-containing surface groups are expected to lower the potentials of zero voltage rise (Φ), while oxygen-containing groups should increase it. This trend has been confirmed by the results shown in Fig. 4. Cyclic voltammetry (CV) profiles of the electrodes are shown in Fig. 4D. Electrodes coated with EDA showed reduced capacitance compared to pristine YP80F. Despite reduced surface areas, electrodes modified with oxidation demonstrated larger capacitance because of the increasing level of surface faradaic activities (Fig. 4D and Fig. S7). Similar results have been obtained for YP50F (Figs. S8 and S9).

### Capacitive mixing mechanism

**Fig. 5.**
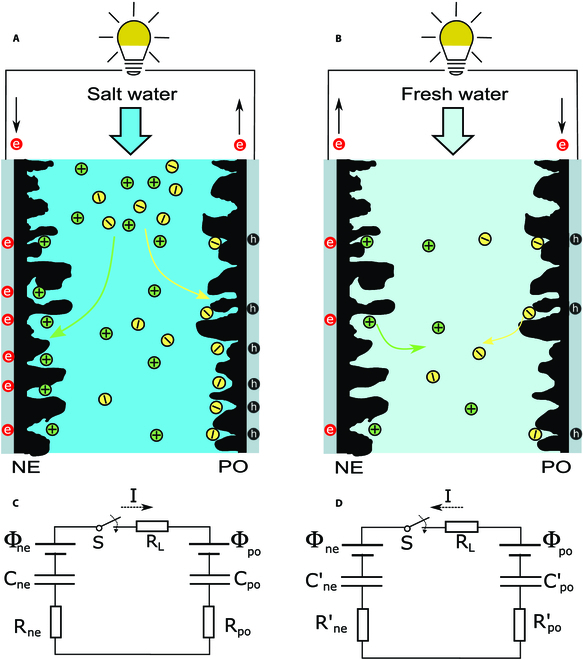
Working principle of generating electricity from salinity gradients. (A) Carbon electrodes immersed in salt water. (B) Carbon electrodes immersed in fresh water. (C and D) Equivalent circuit models for the device in salt water and fresh water, respectively.

Figure 5 shows the working principle of power extraction from a salinity gradient with two electrically neutral electrodes (i.e., *σ*_e_ = 0) containing surface groups with opposite dipoles. The energy harvesting process proceeds in cycles. Without losing generality, we assume the electrodes are firstly immersed in a concentrated solution, i.e., salt water (Fig. 5A); one electrode surface protonates, and the other deprotonates, creating two oppositely charged interfaces. At a high concentration, the magnitude of the Volta potential (*ψ*_0_) is low such that the interfacial potential jump, *χ*, dominates the electrode potential. As a result, the potential of the electrode having negatively charged surface groups (defined as the anode herein) is higher than that containing positively charged surface groups (described as the cathode herein), as shown by the solid lines in Fig. S10A. Closing the circuit equalizes the potentials (dashed lines in Fig. S10A) through an electric current transferring electrons from the cathode to the anode. Each electrode carries a net charge (i.e., *σ*_e_ ≠ 0) after this process. The switch is then opened, and the solution is changed to a diluted one, e.g., fresh water (Fig. 5B). If the EDL expansion outcompetes the reduction of the proton charge, the potential of the anode reduces, and that of the cathode increases (solid lines in Fig. S10B), creating a potential difference. Closing the circuit allows electrons to flow from the anode to the cathode. After the electrode potentials are equalized (dashed lines in Fig. S10B), the switch is opened again, and the solution is switched back to the concentrated one. Suppose the EDL contraction outcompetes the elevated proton charge due to the high concentration. The potential of the anode increases, and that of the cathode decreases (solid lines in Fig. S10A). The device returns to its original state and is ready for the next-cycle power extraction.

The device behavior can be explained with a phenomenological circuit model presented in Fig. 5C and D. Each electrode–electrolyte interface is modeled as a capacitor (e.g., C_ne_) connected in series with a resistor (e.g., R_ne_) and a voltage source (e.g., Φ_ne_). The capacitor represents the total capacitance of the corresponding EDL. The resistor represents the internal resistance of the EDL, which accounts for the effects of both the resistivity of the electrode material and the conductivity of the electrolyte. The constant voltage source represents the potential difference across the interface due to surface dipoles, *χ*. Note that the capacitance and internal resistance vary as the concentration, whereas the voltage source is fixed. The total capacitance corresponding to the high concentration is $C^{h}=C_{\mathrm{ne}}^{h}C_{\mathrm{po}}^{h}/\left(C_{\mathrm{ne}}^{h}+C_{\mathrm{po}}^{h}\right)$, and the total internal resistance is $R^{h}=R_{\mathrm{ne}}^{h}+R_{\mathrm{po}}^{h}$. When the concentration decreases from *c*^h^ to *c*^l^, the surface charge due to (de)protonation of the surface groups can be denoted as $\sigma_{H}^{l}=\beta \sigma_{H}^{h}$. The stored charge of the capacitor after equilibrium is reached at the high concentration is $\sigma_{0}^{h}=C^{h}\Delta \Phi$, where ΔΦ = Φ_ne_ − Φ_po_, and the total surface charge include the existing charge, *σ*_e_ (if any) and $\sigma_{H}^{h}$, i.e., $\sigma_{0}^{h}=\sigma_{e}+\sigma_{H}^{h}$. Here, we assume the electrodes are polarizable; thus, *σ*_e_ does not vary with concentration. When the concentration is changed to *c*^l^, the capacitance is reduced to *C*^l^, and the charge on the capacitor becomes $\sigma_{0}^{l}=\sigma_{e}+\beta \sigma_{H}^{h}$. An open-circuit potential difference arises between 2 electrodes, which is $\Delta U^{l}=\left(C^{h}/C^{l}-1\right)\Delta \Phi +\left(\beta -1\right)\sigma_{H}^{h}/C^{l}$. After closing the electric switch, as shown in Fig. 5F, the device reaches a new equilibrium state. The energy dissipated through the electric load $R_{m}^{l}$ is obtained as $E^{l}=\frac{1}{2}\frac{R_{m}^{l}C^{l}}{R^{l}+R_{m}^{l}}{\left(\Delta U^{l}\right)}^{2}$, where *R*^l^ denotes the internal resistance corresponding to the low concentration. The electric switch is then opened, and the solution is changed back to the high concentration. The change of concentration creates an open-circuit voltage, i.e, Δ*U*^h^ =  − *C*^l^/*C*^h^Δ*U*^l^. A current will flow upon closing the switch. The energy dissipated through the electric load ($R_{m}^{h}$) will be $E^{h}=\frac{1}{2}\frac{R_{m}^{h}C^{l}}{R^{h}+R_{m}^{h}}\frac{C^{l}}{C^{h}}{\left(\Delta U^{l}\right)}^{2}$. In the case where $R_{m}^{l}>>R^{l}$ and $R_{m}^{h}>>R^{h}$, $E^{l}=\frac{1}{2}C^{l}{\left(\Delta U^{l}\right)}^{2}$ and $E^{h}=\frac{C^{l}}{C^{h}}E^{l}$. The energy harvested in the dilute solution is higher than that in the concentrated solution; however, the power generated in the dilution solution will be lower because extracting that amount of energy requires a long time in the dilute solution due to the high impedance. The amount of energy harvested increases quadratically with the voltage rise (e.g., Δ*U*^l^), which is proportional to the surface charge and bias resulting from the surface dipoles. Therefore, manipulating the surface dipoles of otherwise identical electrodes creates an asymmetric electrode surface complexation structure that facilitates salinity-gradient energy harvesting.

### Experiments

As a demonstration, we have used electrodes fabricated with modified YP80F to generate electricity from different salinity gradients, i.e., 0.6 M to 0.01 M, 2 M to 0.01 M, 5 M to 0.01 M, and 5 M to 0.6 M. Electrodes coated with 10% EDA and the ones treated with 8 M nitric acid have been considered. As shown in Fig. 5, an energy extraction cycle included 4 stages: (a) open-circuit potential establishment (high concentration), (b) discharge (high concentration), (c) open-circuit potential establishment (low concentration), and (d) discharge (low concentration).

**Fig. 6.**
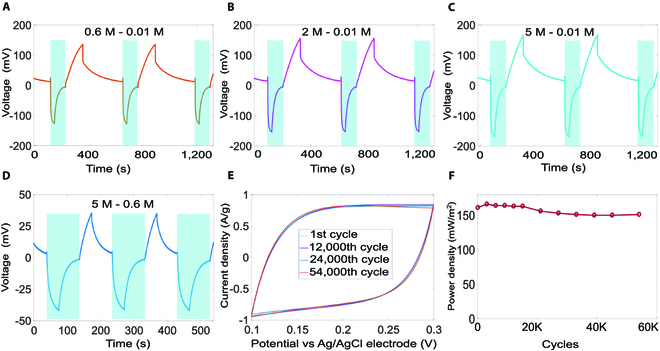
Performance of the prototype. (A to D) Time histories of cell voltage under different salinity gradients. (A) 0.6 M to 0.01 M; harvested energy per cycle: *E* = 19.6 mJ, power density: *P* = 161 mW/m^2^. (B) 2 M to 0.01 M, *E* = 29 mJ, *P* = 236 mW/m^2^. (C) 5 M to 0.01 M, *E* = 38 mJ, *P* = 306 mW/m^2^. (D) 5 M to 0.6 M, *E* = 2.6 mJ, *P* = 58 mW/m^2^. (E) Cyclic voltammetry results of the prototype in 0.6 M NaCl (scan rate: 15 mV/s). (F) Power densities after multiple cyclic voltammetry cycles.

In the 4-stage cycle, mixing takes place in stages 1 and 3, where the bulk solution mixes with the solution in the pores to generate an open-circuit potential. Once mixing (e.g., stage 1) is complete, the solution in the pores has a similar concentration to the bulk solution (high concentration), and the electrical power generated by mixing is extracted in the following discharging stage (e.g., stage 2). Following the completion of discharging, the device is submerged manually in a different bulk solution (e.g., low concentration) to establish a salinity gradient between the bulk and the solution in the pores (Fig. S13), thereby triggering mixing and electricity generation (stage 3). We used the voltage drop method to measure the total internal resistances of the device, and they were 1.3 Ω, 0.8 Ω, 0.6 Ω, and 28.2 Ω in 0.6 M, 2 M, 5 M, and 0.01 M NaCl, respectively. The measured resistances were consistent with the results from electrochemical impedance spectroscopy (Table S3). We used different resistors in Stage 2 and Stage 4 to optimize the total output power density for all but the 5 M to 0.6 M gradient, i.e., 12 Ω and 46 Ω in Stage 2 (0.6 M, 2M, and 5 M) and Stage 4 (0.01 M), respectively. For the 5 M to 0.6 M gradient, one 8.3 Ω resistor was used in both stages. For all salinity gradients, we fixed each cycle to be 540 s, i.e., 30 s, 80 s, 130 s, and 300 s for the 4 stages, respectively. Figure 6A shows that the device produced open-circuit cell voltages of −127 mV and 135 mV in the 0.6 M and the 0.01 M solutions, respectively. The extracted energy was 19.6 mJ/cycle (11 mJ in 0.6 M and 8.6 mJ in 0.01 M), corresponding to an average power density of 161 mW/m^2^. Using the measured internal resistances and the external resistive load, we calculated the total electricity generated from the Gibbs free energy to be 12 mJ in 0.6 M and 14 mJ in 0.01 M, which is consistent with the total capacitance change of 20% calculated using the results of CV experiments. The power density for the seawater stage reached 432 mW/m^2^, with the total power extracted (including that consumed by the internal resistance) being 480 mW/m^2^. For a 2 M to 0.01 M gradient, the voltage reached 153 mV in the 2 M solution and −156 mV in the 0.01 M solution (Fig. 6B), equivalent to a power density of 236 mW/m^2^. Under the hypersalinity condition, i.e., 5 M to 0.01 M, the prototype produced a remarkably high power density of 306 mW/m^2^ (Fig. 6C). We have also tested the prototype with different salinity gradients, including 0.5 M to 0.01 M and 0.5 M to 0.02 M, respectively. For the 0.5 M to 0.01 M gradient, the prototype produced open-circuit cell voltages of −124 mV and 126 mV in the 0.5 M and the 0.01 M solutions, respectively (Fig. S11a). The extracted energy was 16.0 mJ/cycle (9.2 mJ in 0.5 M and 6.8 mJ in 0.01 M), corresponding to an average power density of 130 mW/m^2^. For the 0.5 M to 0.02 M gradient, the prototype produced open-circuit cell voltages of −110 mV and 114 mV in the 0.5 M and the 0.02 M solutions, respectively (Fig. S11b). The extracted energy was 13.5 mJ/cycle (7.2 mJ in 0.5 M and 6.3 mJ in 0.02 M), corresponding to an average power density of 130 mW/m^2^. We have also considered artificial seawater containing multivalent ions, such as magnesium (Mg^2+^), calcium (Ca^2+^), potassium (K^+^), and sulfate (SO${\;}_{4}^{2-}$). In particular, the solution included 0.45 M NaCl, 10 mM KCl, 9 mM CaCl_2_, 30 mM MgCl_2_ · 6H_2_O, and 16 mM MgSO_4_ · 7H_2_O. For the artificial seawater to river water (0.01 M) gradient (Fig. S12), the prototype produced open-circuit cell voltages of −124 mV and 126 mV in the artificial seawater and the river water, respectively. The extracted energy was 15.6 mJ/cycle (9.2 mJ in artificial seawater and 6.4 mJ in river water), corresponding to an average power density of 128 mW/m^2^.

We have studied the long-term performance of the prototype by evaluating its charge–discharge stability. We performed CV in a 0.6 M NaCl solution with a voltage window of 100 to 300 mV and a scan rate of 15 mV/s. Figure 6E shows that the CV curves did not change noticeably after 54,000 charge/discharge cycles. The specific capacitance was initially 41.9 F/g, settling at 40.8 F/g after 54,000 cycles. The peak-to-peak voltage of the device subjected to the 0.6 M to 0.01 M salinity gradient was 262 mV initially; it increased slightly to 268 mV after 3,000 cycles, corresponding to a power density of 166 mW/m^2^, and settled at 265 mV after 54,000 cycles. As shown in Fig. 6F, the power density of the device remained within 90% of the peak value after 54,000 cycles. We also tested the device after it had been stored for 6 months and observed the same performance level.

## Discussion

PRO and RED are the earliest and most studied methods for harvesting the Gibbs free energy from salinity gradients. Because they rely on membranes to maintain the required gradient between the draw solution and the feed solution, performance evaluations have been naturally based on areal metrics. Areal power density, measured in watts per unit membrane area, has been the gold standard to compare membrane-based technologies. In a typical capacitive mixing system, the areal power density is calculated based on the electrode area. The power density values of existing EDL-based membrane-free systems are below 65 mW/m^2^ [29,32,42]. Other technologies, such as battery mixing and hybrid mixing, have been reported to achieve a maximum power density of about 98 mW/m^2^ under a 0.6 M to 0.01 M salinity gradient for membrane-free and charge-free operations [58,59] (Table S5). In contrast, our research has demonstrated that proper surface modifications can increase the power density to 166 mW/m^2^ for a 0.6 M to 0.01 M gradient.

The values of the areal power density of capacitive mixing are generally lower compared to those of the prevailing membrane-based technologies, which can achieve power densities on the order of 2 to 4 W/m^2^ for PRO [13,60] and 0.77 to 1.1 W/m^2^ for RED [60]. However, a direct comparison of these values of capacitive mixing and PRO or RED can be misleading because of the fundamental difference in their working principles and hence their system architecture. From a practical perspective, metrics based on the footprint of essential components seem more suitable for comparing fundamentally different technologies. One such metric, i.e., volumetric power density, has been used for PRO and RED [1,61]. Using the volumes of the carbon materials, we calculated the net volumetric power density of our prototype to be 0.88 kW/m^3^ for a full cycle and 2.3 kW/m^3^ for the seawater stage. Our system’s performance is comparable to PRO (1.1 kW/m^3^) and better than RED (0.16 kW/m^3^) [1,61]. Such high performance in volumetric power density is because capacitive mixing utilizes the pores in the electrodes for mixing. Therefore, it can be highly efficient when the electrode possesses an appropriate 3-dimensional structure. On the other hand, membrane technologies require the draw and feed solutions to be separated by a membrane that enables and regulates mixing. Thus, they need a much larger footprint, resulting in limited volumetric power density. It is noted that caution must be taken when comparing fundamentally different technologies, such as capacitive mixing systems versus PRO or RED because neither existing areal nor volumetric metric is a perfectly suitable performance indicator. Further research is needed to develop a universal performance metric for salinity gradient-based power generation systems.

In sum, electrode surface chemistry is essential for a capacitive mixing system to function without an external charge source. The immobile ionic surface charge, resulting from surface functional groups, regulates electron transfer between the electrodes via external circuitry when the system responds to a salinity gradient. Such electron transfer introduces the same effect as if the electrodes were charged by an external charge source. Therefore, when the salinity gradient is in reverse subsequently, the electrodes exchange their roles, i.e., the cathode behaving as an anode and vice versa. Electrons flow in the opposite direction, allowing the process to progress cyclically. Our study confirms that the performance of such a capacitive mixing system relies on electrochemical effects on both sides of the interface, i.e., the interfacial potential jump on the electrode side and, on the electrolyte side, the change of DEL capacitance induced by the salinity gradient. These effects originate from the surface dipoles, which result from the interactions between surface functional groups and surface atoms. Protonation or deprotonation of the functional groups gives rise to the ionic charge needed for the EDL. The dipoles establish the interfacial potential jump, which is opposite to the Volta potential of the EDL. Interestingly, the Volta potential of the EDL is sensitive to salinity gradient, but the interfacial potential jump is not—a simple explanation is that it is on the electrode side of the interface, on which the solution concentration imposes no influence. Therefore, tuning the surface chemistry modifies the interfacial potential jump directly and affects the EDL indirectly by adjusting the surface charge.

Finally, we note that there are several other avenues to improve the performance of our system further. For example, optimizing the system architecture can increase the power output dramatically by facilitating convection diffusion that can increase the mixing speed by orders of magnitude [62] and, in the meantime, reduce the internal resistance significantly [63], making the technology economically viable. Because of the versatility of surface treatments [64,65], we anticipate that this work will open new doors to designing capacitive mixing systems that will capitalize on their promise in membrane-free operations.

## Materials and Methods

### Materials

Activated carbon materials, YP50F and YP80F, were provided by Kuraray Chemical Co., Ltd. EDA, nitric acid, polyvinylidene fluoride (PVDF), 1-methyl-2-pyrrolidinone (NMP), and carbon black powders were purchased from Sigma-Aldrich Co. Ltd. Graphite foils were acquired from Panasonic Holdings Corporation. Oxygen-containing surface functional groups were introduced by stirring pristine activated carbons in nitric acid at 60 ^o^C for 6 h and constantly washing out with deionized water. Nitrogen-containing surface functional groups were introduced by stirring pristine activated carbons in EDA solutions at 50 ^o^C for 6 h and washing out with deionized water. The carbon samples were collected from water by centrifugation and dried at 60 ^o^C for 48 h.

### Fabrication of carbon electrodes

A Thinky AR-100 mixer was used to mix 0.4 g of modified activated carbon, 0.03 g of carbon black, 0.03 g of PVDF, and 2 g of NMP to obtain a homogeneous slurry. The slurry was coated on a graphite foil current collector by the doctor blade method. Polyethylene terephthalate was used as the substrate for the electrodes. The electrodes were then dried at 60 ^o^C for 48 h.

### Material characterization analysis and electrical measurements

The morphology of carbon materials was examined by Helios 660 NanoLab 660 Dual Beam Focused Ion Beam instrument. Pore size distributions and BET surface areas were measured with a Quantachrome Autosorb-iQ analyzer. Potentiometric acid–base titration experiments were performed using a Metrohm Titrando 888. Aqueous suspensions were prepared by continuously stirring 0.1 g of carbon material in a 90-ml sodium chloride (NaCl) solution with a known amount of sodium hydroxide (NaOH). Three concentrations of NaCl solutions (1 M, 0.1 M, and 0.01 M) were used. The prepared solutions were titrated by adding hydrogen chloride (HCl) under an argon atmosphere. The titration experiments were repeated without carbon materials in the NaCl solutions. Electrochemical measurements, i.e., CV and electrochemical impedance spectroscopy, were conducted with a Gamry Interface 5000E potentiostat. In a 3-electrode configuration, a 1-in^2^ platinum sheet was used as the counter electrode, and an Ag/AgCl electrode with saturated KCl filling solution was used as the reference electrode. Electrical measurements were obtained using a Tektronix MSO44. Electrodes were immersed in a 1 M NaCl solution overnight before experiments. All experiments were conducted at room temperature.

## Data Availability

The data supporting the findings of this study are available within the paper and its Supplementary Materials.
