# Supplementary material for: Tuning Surface Molecular Design of Porous Carbon for Blue Energy Harvesting
Source: Research (Wash D C). 2023 Jun 19;6:0173. doi: 10.34133/research.0173 (PMC10278960; doi:10.34133/research.0173)
Supplement: Supplementary 1 — Figs. S1 to S13 Tables. S1 to S5 [file research.0173.f1.pdf]

Tuning surface molecular design of porous carbon for blue energy harvesting

Jian Yu, Zhong-Lin Wang ,and Tianwei Ma

Supplementary Tables & Figures

Carbon	BET area (m^2/g)	Capacitance(F/g)		Element wt%		
		0.6 M NaCl	0.01 M NaCl	C	N	O
pristine YP50F	1832	35.6	16.7	98.1	0	1.9
0.2% EDA	1686	32.3	15.1	97.21	0.42	2.37
10% EDA	1607	30.1	13.7	95.77	1.45	2.78
0.5 M HNO_3	1760	40.9	17.6	95.44	0	4.56
2 M HNO_3	1591	47.4	18.2	94.34	0	5.66
4 M HNO_3	1574	58.7	18.7	92.09	0	7.91
8 M HNO_3	1564	63.8	22.4	91.39	0	8.61

Table S1. Material properties of activated carbon YP50F with different surface modifications.

Carbon	EIS Internal Resistance(Ω)	
	0.6M NaCl	0.01M NaCl
pristine YP80F	0.5	16
YP80F 0.2% EDA	0.5	16
YP80F 10% EDA	0.4	15
YP80F 0.5M HNO_3	0.3	9
YP80F 2M HNO_3	0.3	10
YP80F 8M HNO_3	0.3	9
YP80F 12M HNO_3	0.4	14

Table S2. Internal resistance of surface-modified YP80F from EIS.

Concentration (mol/L)	EIS internal resistance (Ω)	Voltage drop internal resistance (Ω)
5	0.46	0.6
2	0.57	0.8
0.6	1.07	1.3
0.01	30.3	28.2

Table S3. Electrical properties of the prototype. (One electrode, 47.5 mg, with 8 M HNO_3 modification, and the other, 48 mg, with 10% EDA coating)

Carbon	Micropore Vol. (cm^3/g)	Mesopore Vol. (cm^3/g)	Pore Vol. (cm^3/g)
Pristine	0.54	0.39	0.93
0.2% EDA	0.51	0.31	0.82
10% EDA	0.47	0.27	0.75
0.5 M HNO_3	0.53	0.36	0.89
2 M HNO_3	0.49	0.33	0.82
8 M HNO_3	0.43	0.39	0.82
12 M HNO_3	0.45	0.25	0.70

Table S4. Pore characteristics of activated carbon YP80F with different surface modifications.

Electrodes	Salinity gradient (M)	External power source	Membrane	Average power density mW/m^2	Voltage rise (mV)	tested cycles	Ref.
NMO|AgCl	0.5, 0.02	yes	no	15	120	n/a	[1]
BiOCl|CuHCF	0.5, 0.02	yes	yes	100.1	120	n/a	[2]
BiOCl|CuHCF	0.5, 0.02	yes	no	87	100	50	[2]
AgCl|$LiFePO_4$	0.6,0.02	yes	no	105	100	100	[3]
PPY|FeHCF	0.6, 0.03	no	no	16	130	50	[4]
AC|AC	0.5, 0.02	yes	yes	205	136	n/a	[5]
AC|AC	0.5, 0.01	no	yes	12.9	119	n/a	[6]
AC|AC	0.5,0.02	no	yes	8.4	93	n/a	[7]
AC|AC	0.6,0.02	yes	no	6.6	33	n/a	[8]
AC|AC	0.5-0.6, 0.001-0.02	yes	no	0.13 - 3.3	15.4 - 42.4	n/a	[9, 10, 11, 12]
SWCNT|MWCNT	0.6, 0.01	no	no	24	108	n/a	[13]
AC|NMO	0.6, 0.01	no	no	97	150	n/a	[14]
AC|NMO	0.5, 0.02	yes	no	38.6	110	n/a	[15]
A-PC-2|NS30	0.5, 0.02	no	no	50	53	n/a	[16]
AC-QPVP| AC-HNO_3	0.5, 0.02	no	no	65	150	n/a	[17]
AC-p-TSA| AC-PEI-EN	0.5, 0.02	no	no	28, 35	82, 83	n/a	[18, 1]
AC-PSS| AC-PDADMAC	0.5, 0.02	yes	no	50	120	n/a	[19]
AC-PSS| AC-PDADMAC	0.5, 0.02	no	no	12.1, 30	70, 72	n/a	[20, 21]
YP80F-EDA|FeHCF	0.6, 0.01	no	no	98	-135, 69	150	[22]
YP80F-EDA| YP80F-HNO_3	0.5, 0.02	no	no	121	113	n/a	This work
YP80F-EDA| YP80F-HNO_3	0.6, 0.01	no	no	166	135	54 K	This work

Table S5. Experimental Conditions and Performance of Existing Mixing Devices.

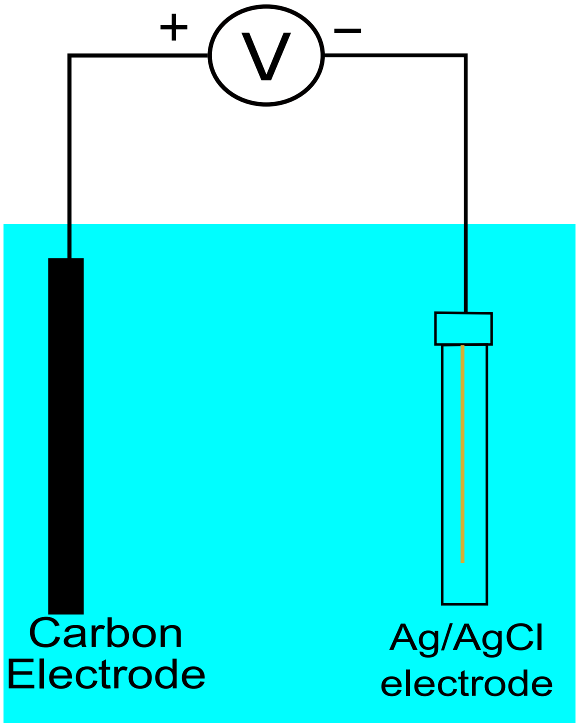

Figure S1. Setup for measuring the spontaneous potential of a carbon electrode versus a Ag/AgCl electrode.

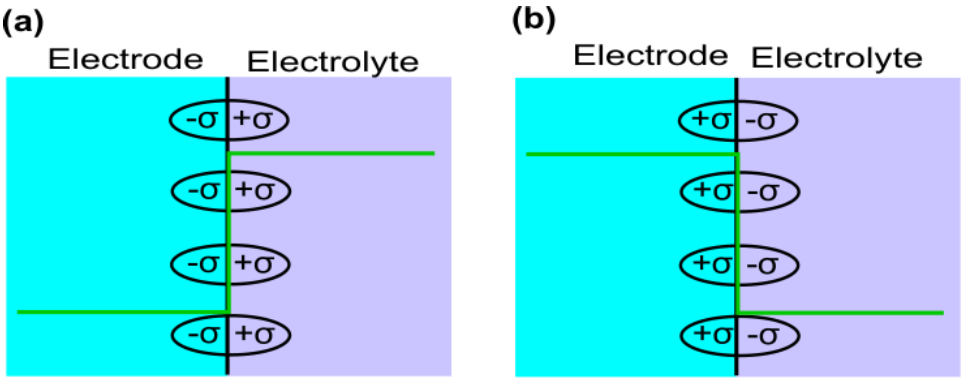

Figure S2. A schematic of the interfacial potential jump, χ (green lines). (a) Dipoles pointing toward the electrode introduce a negative interfacial potential jump from the electrolyte to the electrode. **(b)** Dipoles pointing toward the electrolyte introduce a positive interfacial potential jump from the electrolyte to the electrode.

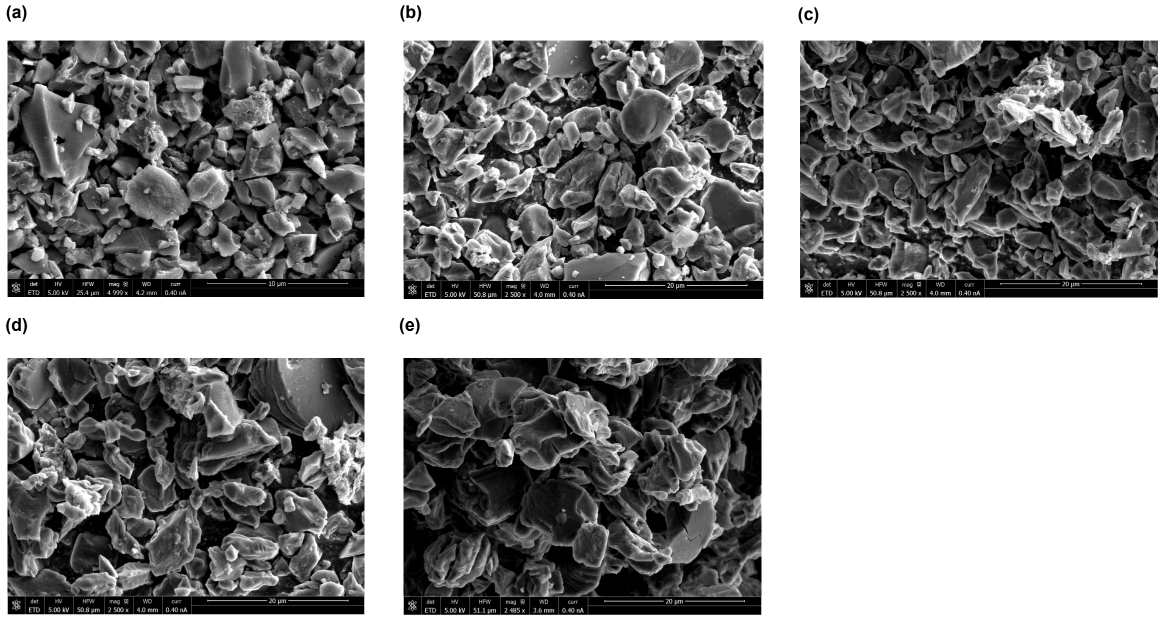

Figure S3. SEM images of YP80F treated with HNO_3. (a) Pristine YP80F. **(b)** YP80F treated with 0.5 M HNO_3. **(c)** YP80F treated with 2 M HNO_3. **(d)** YP80F treated with 8 M HNO_3. **(e)** YP80F treated with 12 M HNO_3.

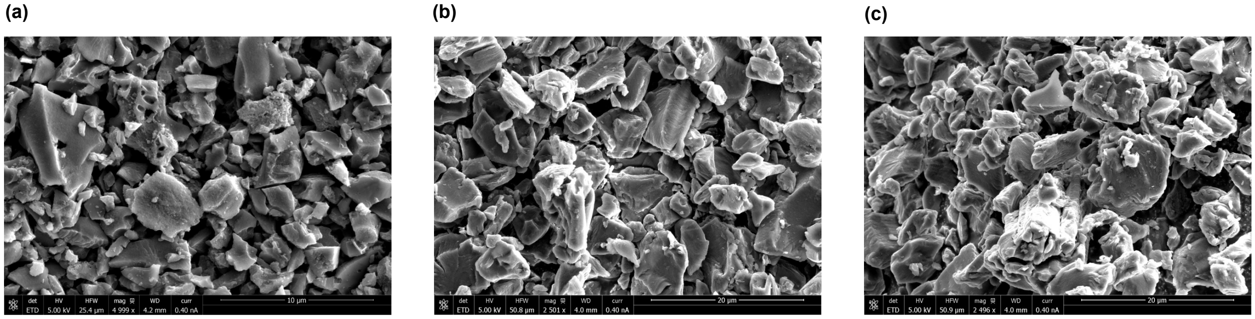

Figure S4. SEM images of YP80F treated with EDA. (a) Pristine YP80F. **(b)** YP80F 0.2% EDA. **(c)** YP80F 10% EDA.

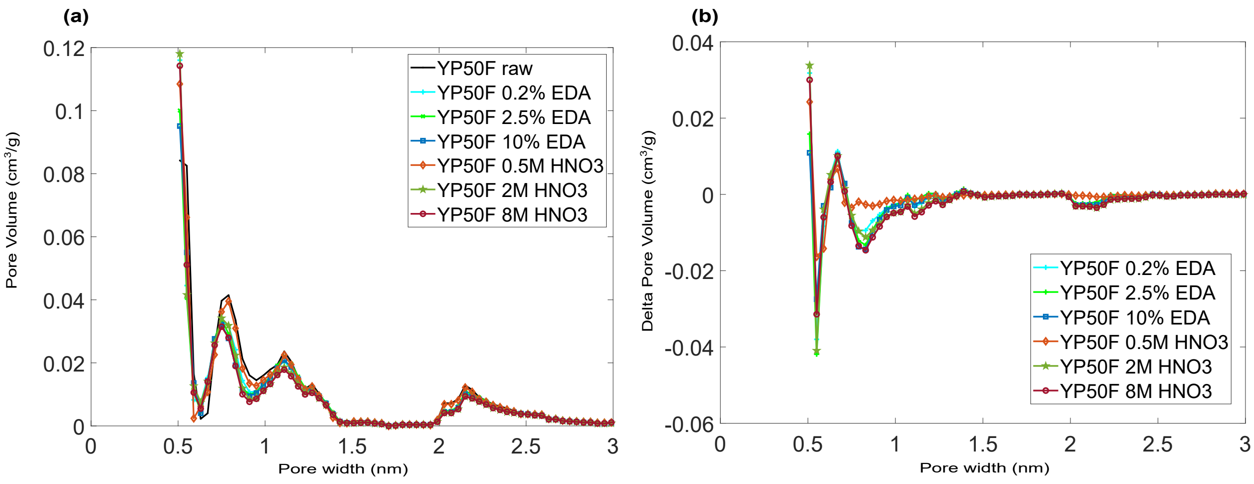

Figure S5. Pore characteristics of surface modified YP50F. (a) Pore size distribution. **(b)** Pore volume differences between surface modified carbons and pristine YP50F.

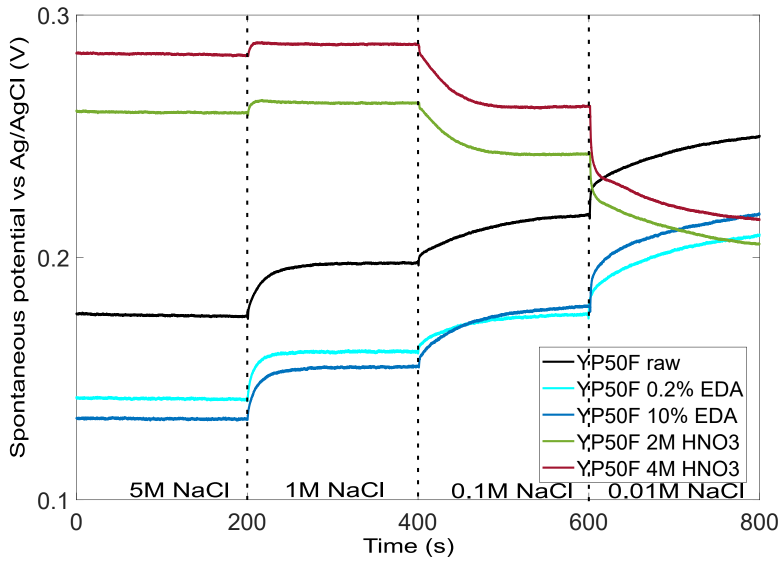

Figure S6. Time histories of electrode potentials under different salinity levels for electrodes fabricated with pristine and surface-modified YP50F

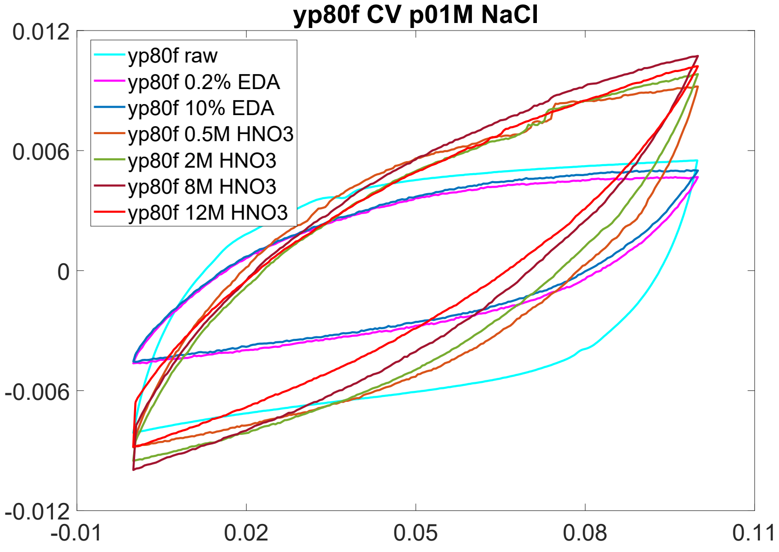

Figure S7. CV results for YP80F in 0.01M NaCl.

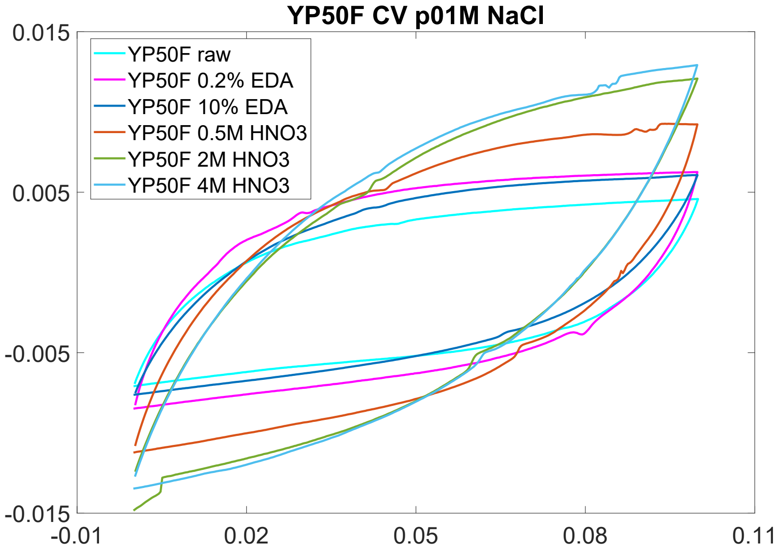

Figure S8. CV results for YP50F in 0.01M NaCl.

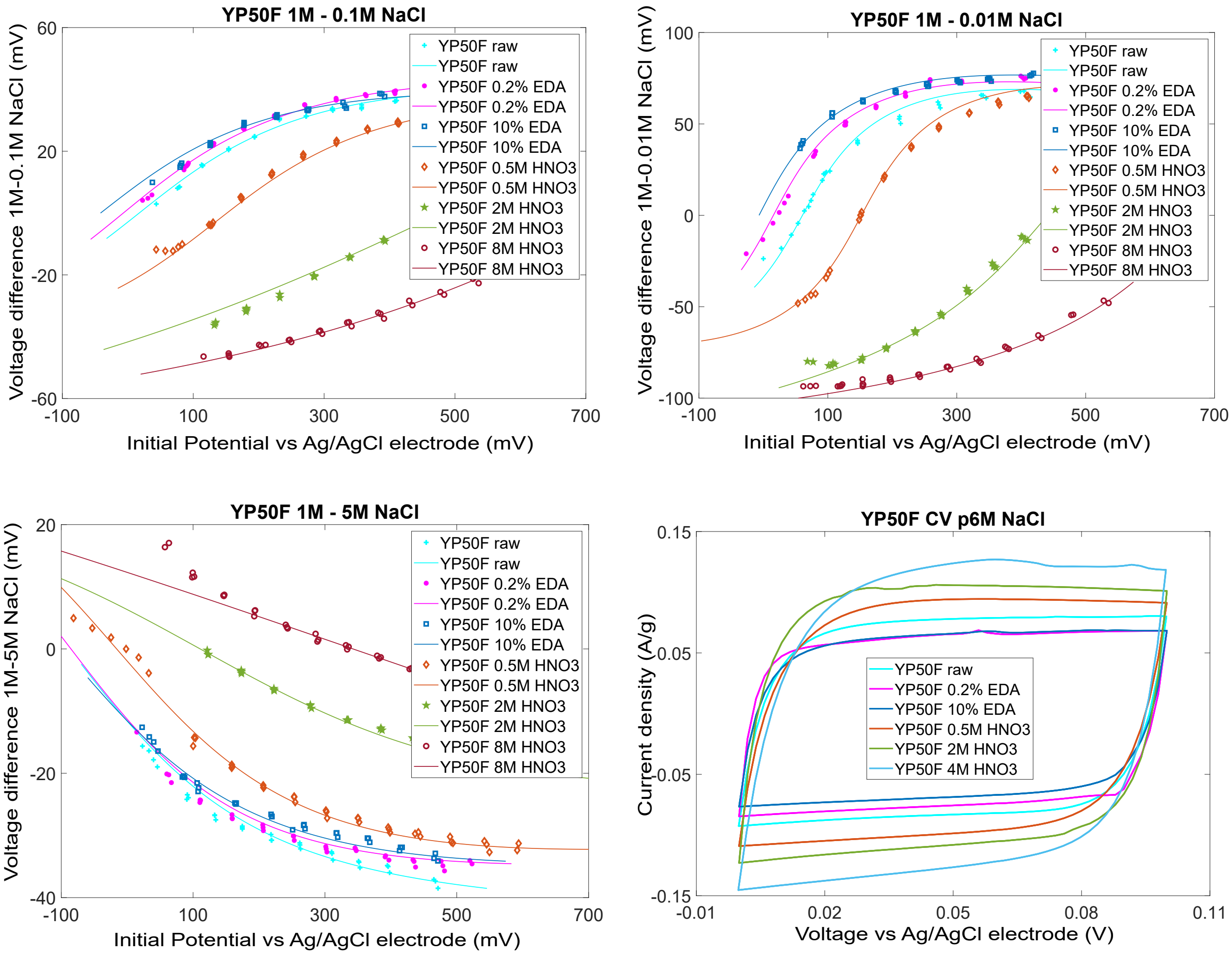

Figure S9. Results obtained from voltage rise and CV experiments for YP50F electrodes.

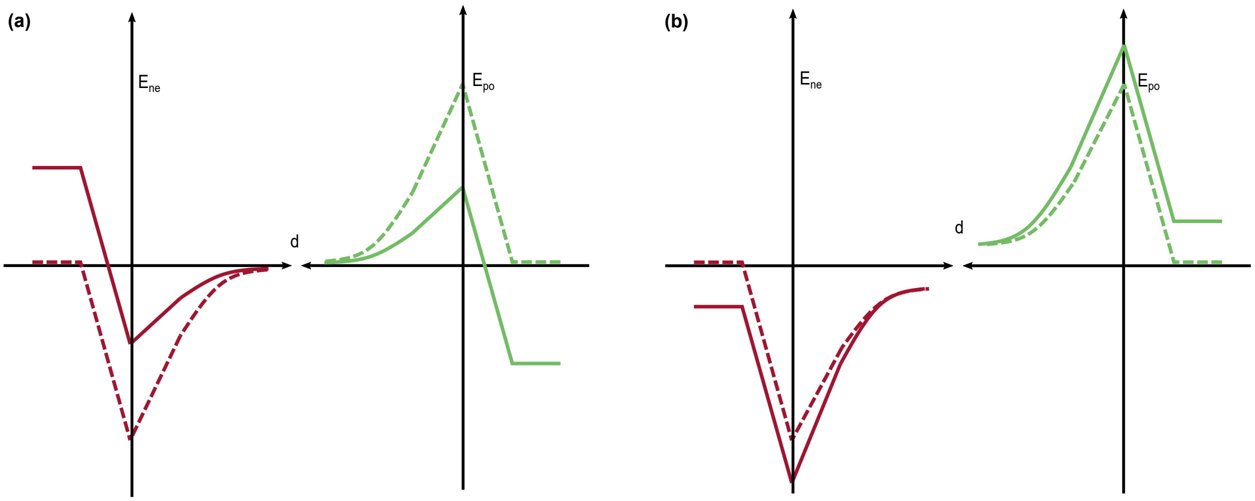

Figure S10. Distributions of electrical potentials of carbon electrodes with different surface properties under a salinity gradient. (**a**) Electrodes in a concentrated solution, and (**b**) Electrodes in a dilute solution. Solid lines: open-circuit potentials. Dotted lines: closed circuit potentials. E_{ne} and E_{po} represents the electrolyte potential with negative-charge and positive-charge surface functional groups, respectively

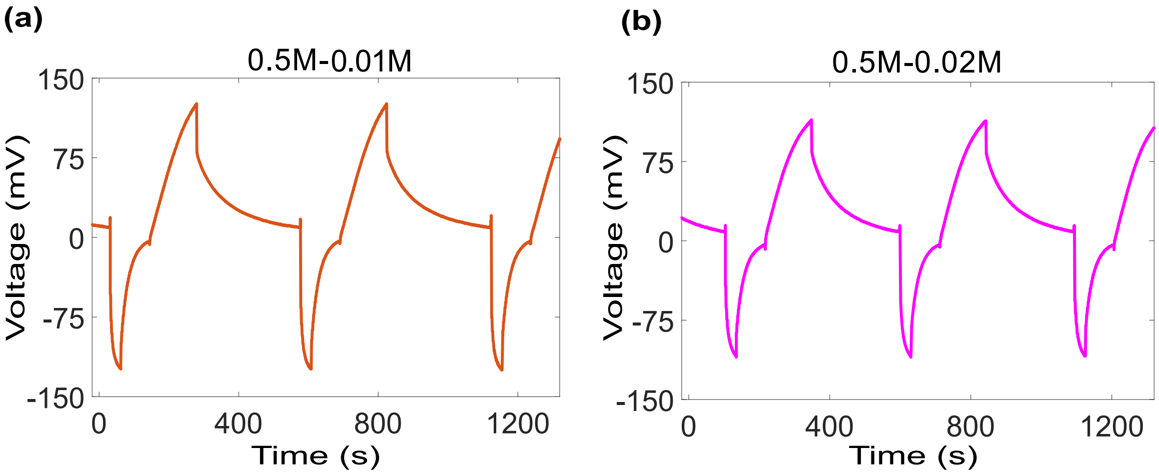

Figure S11. Time histories of cell voltage under different salinity gradients. a. 0.5 M - 0.01 M; harvested energy per cycle: E=16.0 mJ, power density: P = 130 mW/m^2. **b.** 0.5 M - 0.02 M; harvested energy per cycle: E=13.5 mJ, power density: P = 121 mW/m^2.

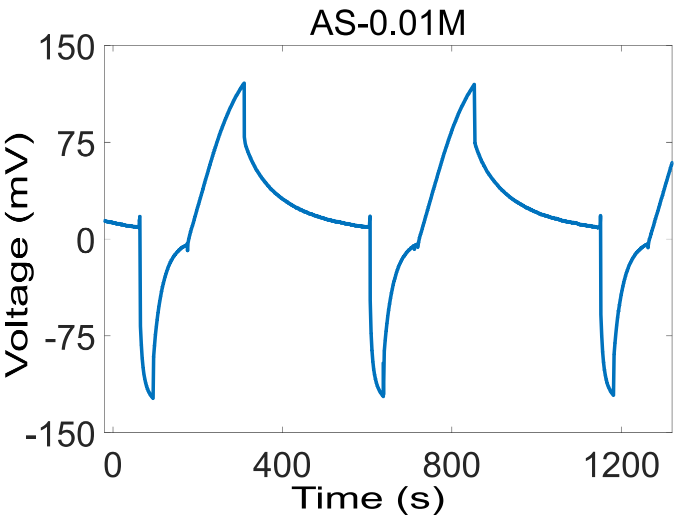

Figure S12. Time histories of cell voltage between artificial seawater and 0.01M NaCl. Harvested energy per cycle: E=15.6 mJ, power density: P = 128 mW/m^2.

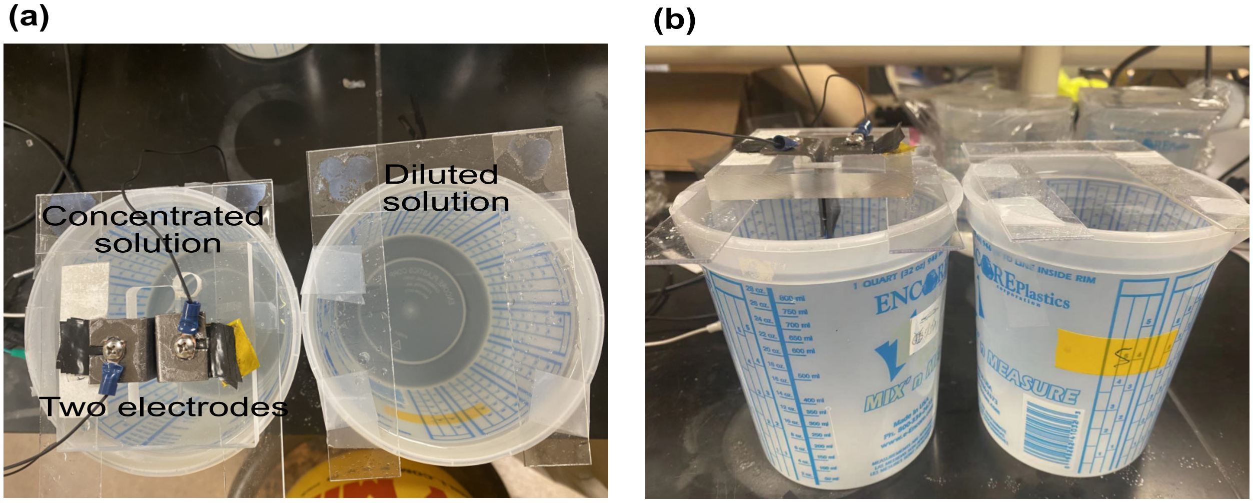

Figure S13. Photos of experimental setup. a. Top view. **b.** Side view.

References

[1] M Marino, L Misuri, R Ruffo, and D Brogioli. Electrode kinetics in the "capacitive mixing" and "battery mixing" techniques for energy production from salinity differences. *Electrochimica Acta*, 176:1065–1073, 2015.

[2] Guangcai Tan and Xiuping Zhu. Polyelectrolyte-coated copper hexacyanoferrate and bismuth oxychloride electrodes for efficient salinity gradient energy recovery in capacitive mixing. *Energy Technology*, 8(1):1900863, 2020.

[3] Fabio La Mantia, Mauro Pasta, Heather D Deshazer, Bruce E Logan, and Yi Cui. Batteries for efficient energy extraction from a water salinity difference. *Nano letters*, 11(4):1810–1813, 2011.

[4] Meng Ye, Mauro Pasta, Xing Xie, Kristian L Dubrawski, Jianqaio Xu, Chong Liu, Yi Cui, and Craig S Criddle. Charge-free mixing entropy battery enabled by low-cost electrode materials. *ACS omega*, 4(7):11785–11790, 2019.

[5] Fei Liu, Olivier Schaetzle, Bruno Bastos Sales, Michel Saakes, Cees JN Buisman, and Hubertus VM Hamelers. Effect of additional charging and current density on the performance of capacitive energy extraction based on donnan potential. *Energy & environmental science*, 5(9):8642–8650, 2012.

[6] BB Sales, M Saakes, JW Post, CJN Buisman, PM Biesheuvel, and HVM Hamelers. Direct power production from a water salinity difference in a membrane-modified supercapacitor flow cell. *Environmental science & technology*, 44(14):5661–5665, 2010.

[7] Bruno B Sales, Fei Liu, Olivier Schaetzle, Cees JN Buisman, and Hubertus VM Hamelers. Electrochemical characterization of a supercapacitor flow cell for power production from salinity gradients. *Electrochimica acta*, 86:298–304, 2012.

[8] Doriano Brogioli. Extracting renewable energy from a salinity difference using a capacitor. *Physical review letters*, 103(5):058501, 2009.

[9] D Brogioli, R Zhao, and PM Biesheuvel. A prototype cell for extracting energy from a water salinity difference by means of double layer expansion in nanoporous carbon electrodes. *Energy & Environmental Science*, 4(3):772–777, 2011.

[10] ML Jiménez, MM Fernández, S Ahualli, G Iglesias, and AV Delgado. Predictions of the maximum energy extracted from salinity exchange inside porous electrodes. *Journal of colloid and interface science*, 402:340–349, 2013.

[11] Guillermo R Iglesias, María M Fernández, Silvia Ahualli, María L Jiménez, Oleksander P Kozynchenko, and Ángel V Delgado. Materials selection for optimum energy production by double layer expansion methods. *Journal of Power Sources*, 261:371–377, 2014.

[12] MM Fernández, S Ahualli, Guillermo R Iglesias, Fernando González-Caballero, Ángel V Delgado, and ML Jimenez. Multi-ionic effects on energy production based on double layer expansion by salinity exchange. *Journal of colloid and interface science*, 446:335–344, 2015.

[13] Jian Yu and Tianwei Ma. Harvesting blue energy with carbon electrodes of asymmetric nanopore distributions. *Nano Energy*, 82:105766, 2021.

[14] Jiho Lee, Hongsik Yoon, Jaehan Lee, Taeyoung Kim, and Jeyong Yoon. Extraction of salinity-gradient energy by a hybrid capacitive-mixing system. *ChemSusChem*, 10(7):1600–1606, 2017.

[15] Fei Zhan, Gang Wang, Tingting Wu, Qiang Dong, Yulan Meng, Jianren Wang, and Jieshan Qiu. Salinity-difference-driven power generation by a hybrid capacitive approach. *Energy Technology*, 6(2):238–241, 2018.

[16] D Brogioli, R Ziano, RA Rica, D Salerno, O Kozynchenko, HVM Hamelers, and F Mantegazza. Exploiting the spontaneous potential of the electrodes used in the capacitive mixing technique for the extraction of energy from salinity difference. *Energy & environmental science*, 5(12):9870–9880, 2012.

[17] Fei Zhan, Gang Wang, Tingting Wu, Qiang Dong, Yulan Meng, Jianren Wang, Juan Yang, Shaofeng Li, and Jieshan Qiu. High performance asymmetric capacitive mixing with oppositely charged carbon electrodes for energy production from salinity differences. *Journal of Materials Chemistry A*, 5(38):20374–20380, 2017.

[18] Massimo Marino, Lorenza Misuri, ML Jiménez, S Ahualli, Oleksei Kozynchenko, Steve Tennison, Marek Bryjak, and Doriano Brogioli. Modification of the surface of activated carbon electrodes for capacitive mixing energy extraction from salinity differences. *Journal of colloid and interface science*, 436:146–153, 2014.

[19] MM Fernandez, RM Wagterveld, S Ahualli, Fei Liu, AV Delgado, and HVM Hamelers. Polyelectrolyte-versus membrane-coated electrodes for energy production by capmix salinity exchange methods. *Journal of Power Sources*, 302:387–393, 2016.

[20] Guillermo R Iglesias, Silvia Ahualli, María M Fernandez, María L Jimenez, and Angel V Delgado. Stacking of capacitive cells for electrical energy production by salinity exchange. *Journal of Power Sources*, 318:283–290, 2016.

[21] Silvia Ahualli, ML Jimenez, Marıa M Fernández, Guillermo Iglesias, Doriano Brogioli, and AV Delgado. Polyelectrolyte-coated carbons used in the generation of blue energy from salinity differences. *Physical Chemistry Chemical Physics*, 16(46):25241–25246, 2014.

[22] Bin Yang, Jian Yu, and Tianwei Ma. A charge-free and membrane-free hybrid capacitive mixing system for salinity gradient energy harvesting. *J. Mater. Chem. A*, 11:3388–3398, 2023.
